# Editorial: The role of calcium channels in human health and disease—Volume II

**DOI:** 10.3389/fmolb.2023.1180456

**Published:** 2023-03-21

**Authors:** Peng Zhang, Chang-Bo Zheng, Zhen Chen, Xiao-Yu Liu

**Affiliations:** ^1^ Shenzhen Key Laboratory of Otorhinolaryngology, Shenzhen Institute of Otorhinolaryngology and Longgang Otorhinolaryngology Hospital, Shenzhen, China; ^2^ School of Pharmaceutical Science and Yunnan Key Laboratory of Pharmacology for Natural Products, Kunming Medical University, Kunming, China; ^3^ Winship Cancer Institute, Emory University, Atlanta, GA, United States; ^4^ School of Medicine, Shenzhen Middle School, Southern University of Science and Technology, Shenzhen, China

**Keywords:** calcium channels, calcium signal, human health, human disease, therapeutic target

Calcium is among the most important second messengers in the cells and participates in multiple physiological and pathological progress (Baird GS, 2011). The extracellular calcium concentration (about 1–2 mmol/L) is much higher than the cytoplasmic concentration (about 100–200 nmol/L) in resting condition, and more than 90% of cytoplasmic calcium is stored in intracellular calcium pools (endoplasmic reticulum and mitochondria) (Matikainen et al., 2021). Calcium channels mediate the calcium influx or calcium release from intracellular pools in response to various stimuli. Abnormal calcium homeostasis is associated with various pathologic disorders, including cardiovascular diseases, cancer, immune diseases, and neurological disorders (Berridge MJ, 2012). Therefore, understanding how calcium channels regulate calcium homeostasis is important for identifying novel therapeutic targets. This Research Topic entitled “*The Role of Calcium Channels in Human Health and Disease—Volume II*” features the critical role of calcium channels in regulating human health and disease. The six studies included in this special Research Topic focus on the novel roles of calcium channels in pathophysiological processes.

The transient receptor potential (TRP) channel subfamilies include TRPA, TRPC, TRPM, TRPV, TRPN, TRPP, and TRPML. Members of these subfamilies can be activated by heat, mechanical force, hormones, and osmotic pressure (Nilius et al., 2005; Vangeel and Voets, 2021). TRP channels are widely expressed in the excitable and non-excitable cells, where they regulate cell contraction, secretion, sensory transduction, differentiation, migration, proliferation, and death. Four original research papers in this Research Topic focused on the regulatory role of TRP channels in human cancers. Zhang et al. reported that TRPC7, TRPV4, TRPM2, TRPM4, and TRPM8 were highly expressed in ovarian cancer and negatively correlated with the overall survival period of the patients. They also found that TRPV4 could be used as a biomarker and therapy target for ovarian cancer. Furthermore, Wang et al. demonstrated that TRPV1 expression was downregulated in cervical squamous cell carcinoma, and the expression positively correlated with the overall survival and disease-specific survival of patients. This study suggested that TRPV1 was positively correlated with T cell immune infiltration and negatively correlated with NK cells. Moreover, GO and KEGG analyses showed that TRPV1 plays a key role in ferroptosis and sequestering of metal ions in cervical squamous cell carcinoma, providing insights for further investigations. Wang et al. explored the role of TRPM4 and TRPV2 in uveal melanoma using bioinformatics techniques. They demonstrated that TRPM4 and TRPV2 levels were higher in uveal melanoma and the expression of TRPM4 and TRPV2 were negatively correlated with the prognosis of uveal melanoma patients. Additionally, the expression of TRPV2 and TRPM4 were negatively correlated with ferroptosis pathway relative genesSAT1, RPL8, and GPX4. Zhang et al. found that TRPV4 mRNAs and protein levels were increased in nasopharyngeal carcinoma cells and clinical tissues. However, inhibiting the expression or function of TRPV4 suppressed the nasopharyngeal carcinoma cell growth through NFAT4 inactivation *via in vitro* and *in vivo* studies. Thus, the study suggested that the TRPV4-NFAT4 pathway may be a potential therapeutic target for nasopharyngeal carcinoma treatment.

One review paper in this Research Topic focused on the role of TRP channels in regulating human endocrine-related diseases. Liu et al. gave an outstanding review of the regulatory mechanisms of TRP channels in the pancreas, salivary gland, adrenal gland, mammary gland, gallbladder, sweat glands, and lacrimal gland. They suggested that drug discovery targeting TRP channels would be an effective strategy for treating human endocrine-related diseases.

Orai1 is a subunit of Ca^2+^ release-activated Ca^2+^ (CRAC) channels mediating store-operated Ca^2+^ entry (SOCE), which elevates the cytoplasmic Ca^2+^ level in various cell types. Orai1-mediated SOCE is important for cellular function and pathological processes. One original research paper in this Research Topic explored the role of CRAC channels in regulating cervical cancer. Pan et al. highlighted that Orai1 is overexpressed in cervical cancer tissues and Orai1-mediated SOCE/IL-6 signaling pathway, is the key mechanism underlying cervical cancer cell growth. They suggested that Orai1 would be a therapeutic target for cervical cancer.

In conclusion, the articles included in this Research Topic provide insight into TRPs and CRAC channels implicated in human health and disease. With the rapid development in structural biology, novel drugs targeting calcium channels will be developed to treat human diseases.
